# Can Ectoparasite Phylogenetics Shed Light on Host Evolution? The *Batracobdella* Leeches and *Speleomantes* Salamanders' System

**DOI:** 10.1002/ece3.73019

**Published:** 2026-02-15

**Authors:** María Torres‐Sánchez, Michael Veith, Enrico Lunghi

**Affiliations:** ^1^ Department of Life, Health, and Environmental Sciences University of L'Aquila L'Aquila Italy; ^2^ Biogeography Department Trier University Trier Germany

**Keywords:** amphibian leeches, *Batracobdella*, co‐evolution, co‐phylogeny, Fahrenholz's rule, host–parasite interactions, *Hydromantes*

## Abstract

Understanding species' evolutionary history is essential for comprehending trait diversity. Evolutionary relationships among many species, however, have conflicted phylogenetic inferences due to evolutionary discordances and need additional sources of information. Phylogenetic relationships of symbionts can inform about the evolution of their hosts. In this study, we reconstructed the evolutionary relationships among Sardinian salamander ectoparasitic leeches to resolve evolutionary discordances among their hosts, the Sardinian *Speleomantes* salamander species, and proposed novel evolutionary hypotheses that account for host–parasite evolutionary processes. We inferred the most up‐to‐date phylogeny for *Batracobdella* leeches and uncovered high levels of genetic diversity in *B. algira*, a species commonly known as the amphibian leech, revealing three divergent lineages. Leech phylogeny complemented previous information about Sardinian *Speleomantes* evolutionary history. Our comparison of this parasite phylogeny with the most recent inferred salamander tree revealed potential processes of host–parasite co‐evolution and a host‐switching event in the studied system. In both trees, a clade comprising species with the most northeastern distributions in Sardinia ( *S. flavus* , *S. imperialis* , and *S. supramontis* and their respective leeches) was recovered. The phylogenetic position of the *S. genei* ectoparasitic leech was incongruent with that of its host in previous studies, which recovered *S. genei* as sister to the other Sardinian *Speleomantes*. Disentangling the evolutionary processes underlying these host–parasite interactions is important for understanding not only the evolution of these parasitic leeches but also that of their amphibian hosts.

## Graphical abstract

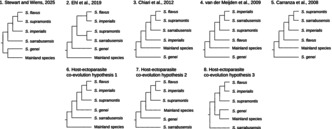

## 1 Introduction

Knowledge about evolutionary relationships among species is crucial for comparative biology. Species can share traits due to their common evolutionary history and ancestry (Felsenstein 1985; Harvey and Purvis 1991). When studying variation across species, the species' evolutionary relatedness should be considered to disentangle trait diversity. The evolutionary relationships among many organisms are, however, still awaiting description or lack well‐supported phylogenetic hypotheses. Understudied organisms represent evolutionary knowledge gaps, but even species groups that have been previously investigated can present conflicts and discordances in their inferred evolutionary history (Kapli et al. 2021; Steenwyk et al. 2023). For instance, burst evolutionary events (i.e., rapid species diversifications) typically result in short branches and clades with low support values on the inferred phylogenies, leading to unresolved evolutionary relationships. Advances in sequencing technologies, which generate high‐throughput sequencing data (i.e., genomic data), and refined methodological approaches have enabled the resolution of phylogenetic relationships across the tree of life (Williams et al. 2020). Nevertheless, many species lack genomic records as these data remain costly to produce and analyse (e.g., species with large repetitive genomes, such as salamanders) (Kosch et al. 2024). Therefore, uncertain evolutionary relationships could greatly benefit from other sources of information, such as symbiont phylogenies. Closely related symbionts often interact with closely related hosts due to co‐speciation and co‐evolutionary processes, thereby presenting congruencies in the reconstructed evolutionary histories of hosts and symbionts (Hayward et al. 2021). Regarding a special type of symbiotic relationship, parasitism, Fahrenholz's rule states that host and parasite phylogenies should be topologically identical (Fahrenholz 1913; Hafner and Nadler 1988). Given the ubiquity of symbiotic interactions in nature (Margulis 1998), insights into the evolution of symbionts could help to enlighten the evolution of their hosts.

Among the tetrapod groups with unresolved evolutionary history are the European plethodontids. Plethodontids are the most diverse group of salamanders (family Plethodontidae Gray, 1850; 523 species; Amphibian Species of the World 6.2 was accessed on 30 September 2025). This family has a disjunct distribution, being currently almost exclusively distributed in the Nearctic and Neotropical regions, but having also a few representatives in the Palearctic region (one species in Asia, *Karsenia koreana* Min, Yang, Bonett, Vieites, Brandon, and Wake, 2005, and eight species in Europe, genus *Speleomantes* Dubois, 1984) (Shen et al. 2016; Stewart and Wiens 2025). The only documented radiation event outside the Americas gave rise to the *Speleomantes* salamanders, a facultative cave‐dwelling plethodontid group commonly known as the European cave salamanders (Lanza et al. 2005). This evolutionary distinct group diverged from its sister genus *Hydromantes* Gistel, 1848, which is distributed in western North America, during the late Oligocene, following the burst emergence of the main groups of the subfamily Plethodontinae Gray, 1850 (Stewart and Wiens 2025). Many phylogenetic relationships within *Speleomantes* and between the clade formed by *Speleomantes* and *Hydromantes* and other Plethodontinae genera (e.g., the genus *Ensatina* Gray, 1850) remain unresolved.

*Speleomantes* are composed of three species distributed in mainland Europe (Italy, San Marino, and France) and five endemic species of the Mediterranean island of Sardinia, where they present allopatric distributions with narrow ranges (four species distributed in the east of the island and one in the southwest) (Lanza et al. 2005). Overall, mainland *Speleomantes* have congruent and well‐supported inferences of their phylogenetic relationships: *S. strinatii* (Aellen, 1958) is the sister group of the clade formed by *S. ambrosii* (Lanza, 1955) and *S. italicus* (Dunn, 1923) (Stewart and Wiens 2025). Yet, the divergence of this mainland clade from the Sardinian *Speleomantes* and the evolutionary relationships among the island species remain controversial. Sardinian *Speleomantes*' evolutionary relationships have been studied multiple times, using from allozymes to a maximum of five mitochondrial genes and one nuclear marker, resulting in phylogenetic inferences with several evolutionary conflicts and discordances (Carranza et al. 2008; Chiari et al. 2012; Ehl et al. 2019; Nascetti et al. 1996; Stewart and Wiens 2025; van der Meijden et al. 2009). Additional evidence of the diversification of this group derives from studies of the species' cytogenetics and endoparasite composition (i.e., presence of sexual chromosomes and presumably the same helminth parasites in mainland and eastern Sardinian species) (Lanza et al. 1995; Nardi et al. 1986). Several species of endoparasites and ectoparasites have been described for *Speleomantes* species (Lanza et al. 2005). Interestingly, an ectoparasitic leech of the genus *Batracobdella* Viguier, 1897 (family Glossiphoniidae Vaillant, 1890) has been found parasitising only Sardinian *Speleomantes* (Lanza et al. 2005; Lunghi et al. 2018; Manenti et al. 2016) and has been ascribed, although with no genetic evidence, to the species *B. algira* (Moquin‐Tandon, 1864) (Lanza et al. 2005). This leech, commonly known as the amphibian leech, was first described as a freshwater leech and has been recorded feeding on several amphibian hosts along the Mediterranean rim (Ben Ahmed et al. 2009, 2015).

**FIGURE 1:**
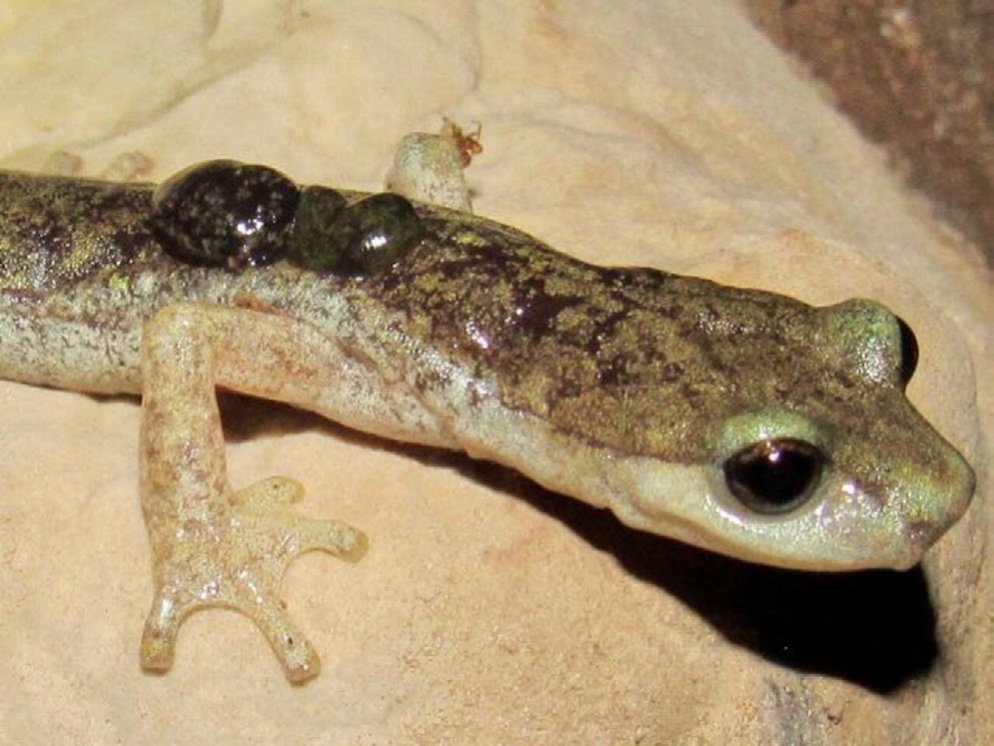
The *Batracobdella* leeches and *Speleomantes* salamanders' system. Photo capturing two *Batracobdella* leeches attached in the back of a *Speleomantes* salamander (credit to MT‐S).

Here, we studied the phylogenetic relationships of Sardinian salamander ectoparasitic leeches to explore Fahrenholz's rule in this host–parasite system (Figure 1). By comparing the topologies of hosts and parasites using the most recent salamander phylogeny, we aimed to resolve discordances in host evolutionary history and to propose novel evolutionary hypotheses for *Speleomantes* salamanders, accounting for host–parasite co‐evolutionary processes. At the same time, we characterised for the first time Sardinian salamander ectoparasitic leeches at the molecular level and inferred their evolutionary relationships using publicly available sequences of *B. algira* and other species of the genus *Batracobdella*.

## 2 Material and Methods

Sampling was focused on collecting ectoparasitic leeches (genus *Batracobdella*) from the five Sardinian cave salamander species, the southwestern Sardininan species: *Speleomantes genei* (Temminck and Schlegel, 1838), and the eastern Sardininan species: *S. flavus* (Stefani, 1969), *S. imperialis* (Stefani, 1969), *S. supramontis* (Lanza, Nascetti, and Bullini, 1986), and *S. sarrabusensis* Lanza, Leo, Cimmaruta, Caputo, and Nascetti, 2001. We surveyed a total of 11 *Speleomantes* populations for which previous information on the presence of parasitic leeches was available (Lunghi et al. 2018; Manenti et al. 2016). Parasitised salamanders were captured to collect the leeches attached to them. We detached the leeches from their salamander host using forceps and stored them in tubes containing pure ethanol. Three additional samples were obtained from a parasitised Corsican brook salamander ( *Euproctus montanus* [Savi, 1838]) and two free‐living leeches (species from the family Erpobdellidae Blanchard, 1894), one collected in a Sardinian stream and the other in a cave in mainland Italy (Stiffe's cave, Abruzzo, Italy). From these samples, we isolated DNA and amplified two mitochondrial barcode regions: the cytochrome C oxidase subunit I (COI) and the 16 small subunit ribosomal RNA gene (16S), using universal versatile primers (Palumbi et al. 1991). Amplified products were Sanger sequenced.

After sequencing, the trace files were visually inspected using SnapGene v8.1.1 (from Dotmatics; available at snapgene.com). We *de novo* assembled forward and reverse overlapping Sanger reads for each barcode of each leech sample to obtain contiguous sequences, using the CAP3 assembler implemented in SnapGene. The low‐quality ends of sequences were automatically trimmed before running CAP3. Some of the sequences had overall low base‐calling quality and could not be assembled (two for the COI and eight for the 16S). We evaluated and manually curated assembled sequences by exploring original sequence chromatograms to validate mismatches, gaps, and insertions. We used BLAST (megablast tool) to confirm the barcode identity of each assembled sequence and to characterise the percentage of similarity between our query sequences and their best hits in the NCBI non‐redundant nucleotide database (Altschul et al. 1990). The assembled sequences were grouped by barcode to create two datasets (COI and 16S datasets) for subsequent phylogenetic analyses. We included in each dataset all the publicly available NCBI GenBank sequences for each barcode of the genus *Batracobdella* (the NCBI GenBank database was accessed on 4 August 2025). Accordingly, in addition to the newly generated sequences, the COI dataset contained sequences for four *Batracobdella* species: *B. algira*, *B. cancricola* Oka, 1928, *B. paludosa* (Carena, 1824), and *B. reticulata* (Kaburaki, 1921), while the 16S dataset included sequences from only one additional *Batracobdella* species: *B. paludosa* .

To investigate phylogenetic relationships of the Sardinian salamander ectoparasitic leeches, we first built multiple sequence alignments for each dataset using the program MAFFT v7 (Katoh and Standley 2013). Given the global expected homology of the sequences in each dataset, we aligned them using the iterative refinement method G‐INS‐i, adjusting sequence direction to match the first sequence in each dataset. For the two multiple sequence alignments, we identified the best‐fitting nucleotide substitution model using the program jModelTest v2.1.10 with the corrected Akaike Information Criterion for model selection (Darriba et al. 2012). We inferred phylogenetic relationships using maximum likelihood with RaxML‐NG v1.2.2 (Kozlov et al. 2019). Phylogenetic inferences were performed using 20 starting trees (10 parsimonious and 10 random trees) with TIM2 + I + G and TVM + G evolutionary models applied to the COI and 16S datasets, respectively, and the free‐living leech sequences used as outgroups. Branch support for the best trees was calculated using 10,000 non‐parametric bootstrap replicates (bootstrapping converged at 1200 replicates for the COI dataset). We associated and compared the pruned COI best tree containing the salamander ectoparasitic *Batracobdella* species with the most recent tree of life of their salamander host species (Stewart and Wiens 2025). We represented and annotated trees using the R package phytools (Revell 2024). To support this comparison, we computed the Shimodaira‐Hasegawa test between the host and parasite tree topologies (with equal edge weights), using a subset of the parasite COI alignment with a representative parasite sequence per host species (see Table S1) and 1 million bootstrap replicates with the R package phangorn (Schliep 2011).

## 3 Results

We collected a total of 21 leeches, of which 19 were salamander ectoparasites (18 from Sardinia and one from Corsica) and two were free‐living animals (see Table S1). The ectoparasitic *Batracobdella* leeches from Sardinia were unevenly found across salamander hosts: nine in *S. flavus* from three populations, four in *S. imperialis* from three populations, three in *S. sarrabusensis* from one population, one in a population of *S. supramontis* , and one in a population of *S. genei* . Some amplified barcode products of these leeches were not properly sequenced, resulting in genetic datasets with fewer than 21 new samples (see Table S1). The mean length of the newly generated sequences was 695 and 534 bases for COI and 16S sequences, respectively. The newly generated COI sequences of the Sardinian salamander ectoparasitic leeches were homologous to a COI sequence from *B. algira* (GenBank ID: OR366856) with 91% identity on average (ranging from 90.61 to 91.78), with this sequence being the best target hit of the BLAST searches. The top homologous COI sequence of the Corsican salamander ectoparasitic leech was also a COI sequence from *B. algira* (GenBank ID: OR366855) with 92.17% of identity. On the other hand, the COI sequences of the two free‐living leeches from Sardinia and mainland Italy were homologous to COI sequences from species of the genus *Dina* Blanchard, 1892 with 89.09% and 96.5% identity, respectively (GenBank ID of the best BLAST hit: MG949122 and GenBank ID of the best BLAST hit: MW459642). At the time of this analysis, there were no 16S sequences available for *B. algira* in the NCBI GenBank database, resulting in the complete mitogenome from *B. cancricola* (GenBank ID: NC_072220) being the best hit for the 16S sequences of the Sardinian salamander ectoparasitic leeches (90% identity on average). The 16S sequences of the free‐living leeches from Sardinia and mainland Italy had sequences from *Dina* and *Erpobdella* species as best BLAST hits (GenBank IDs: MT573325 and NC_036150 with 85.86% and 89.98% identity, respectively).

**FIGURE 2:**
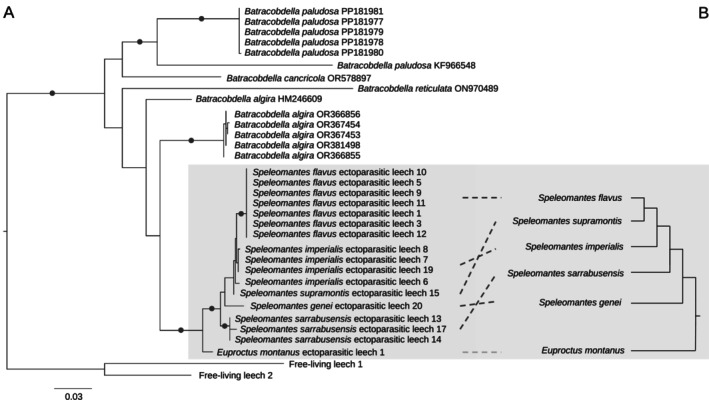
Host‐ectoparasite co‐phylogeny. (A) Inferred COI phylogeny for *Batracobdella* leeches (bullets represent clades with > 70 bootstrap support). (B) Topology of the most recent inferred salamander phylogeny. Grey box highlights the *Speleomantes*‐*Batracobdella* co‐phylogeny with dashed lines indicating the position of host species.

**FIGURE 3:**
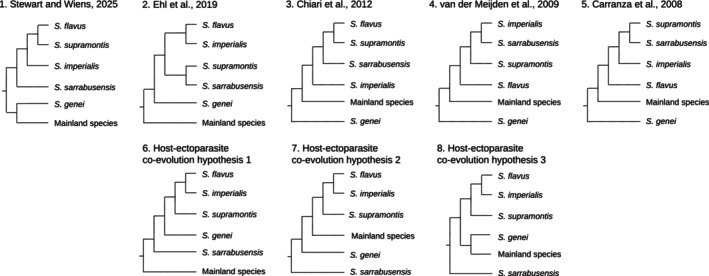
Evolutionary hypotheses of the relationships among the European cave salamanders (*Speleomantes*). 1 to 5 represent the topologies of the inferred phylogenetic relationships among *Speleomantes* species from previous studies. 6 to 8 show the hypothesised topologies based on the inferred phylogeny of the Sardinian salamander ectoparasitic leeches.

The evolutionary relationships among samples were represented by the inferred trees (Figure 2A and Figure S1 for COI and 16S, respectively). All *Batracobdella* sequences (both from NCBI GenBank and from the samples generated in this study) clustered together, forming a clade with 100% bootstrap support. In the COI tree, the newly generated sequences of the salamander ectoparasitic leeches comprised an independent clade (97% bootstrap support), with its sister group being a clade containing most of the *B. algira* NCBI GenBank sequences. This salamander ectoparasitic leech clade contained a clade with the Sardinian leeches. The Sardinian leech clade was supported by a bootstrap value of 86. Within this Sardinian leech clade, the sequences from *S. sarrabusensis* formed an independent clade (98% bootstrap support), being the sister group of all the other Sardinian salamander ectoparasitic leeches. The first split in this later group led to the external node of the *S. genei* ectoparasitic leech and a clade with short bifurcating branches, where *S. supramontis* ectoparasitic leech was the sister node of the clade grouping *S. imperialis* and *S. flavus* ectoparasitic leeches. While *S. imperialis* ectoparasitic leeches did not constitute a monophyletic group, *S*. *flavus* ectoparasitic leeches were grouped together (95% bootstrap support). Similar phylogenetic relationships and topology were recovered in the 16S tree, where *S. imperialis* and *S. flavus* leeches were sister clades (Figure S1). To compare the evolutionary relationships of the salamander ectoparasitic leeches with their hosts, we used the topology of the most recent salamander phylogenetic tree, mirroring the salamander ectoparasitic leech COI subtree in a co‐phylogeny (Figure 2B). Two inverted phylogenetic positions were detected between *S. supramontis* and *S. imperialis* , and between *S. sarrabusensis* and *S. genei* , leading to the proposal of new evolutionary hypotheses of the relationships among *Speleomantes* species (Figure 3). The Shimodaira‐Hasegawa test indicated that the host tree topology fitted the COI parasite alignment data (*p*‐value > 0.05, see Table S2).

## 4 Discussion and Conclusion

In this study, we reconstructed, for the first time, the evolutionary history of the Sardinian salamander ectoparasitic leeches and their relationships with other *Batracobdella* leeches, yielding the most complete phylogeny of this ectoparasitic leech genus to date. These analyses uncovered high levels of genetic diversity among samples identified as *B. algira*. We also investigated the association between the phylogeny of this amphibian parasite and the phylogeny of their amphibian hosts in an unprecedented co‐phylogenetic analysis, pioneering in the exploration of Fahrenholz's rule for the *Batracobdella* leeches and *Speleomantes* salamanders' system. The comparison between host and parasite phylogenies revealed some congruences in the evolutionary history of *Batracobdella* leeches and *Speleomantes* salamanders in Sardinia, and accordingly, potential co‐evolutionary processes. This ectoparasite phylogeny provides complementary evidence of the evolutionary relationships among their *Speleomantes* salamander hosts, with limitations related to the small sample size for some host species.

*Batracobdella* species are mainly freshwater leeches that parasitise numerous animals worldwide, including many amphibian species. An early taxonomic review of this genus recognises a total of 16 species, designating *B. algira* as the type species of the genus (Soós 1967). Since then, many other *Batracobdella* species have been discovered, and others have been relocated into different genera, or both, for example, *Placobdella cryptobranchii* (Johnson and Klemm 1977), which was first described as *B. cryptobranchii* (Johnson and Klemm 1977; Moser et al. 2013). Despite this, the systematics of this group still await formal revision. The phylogeny inferred in our study reflects the most complete evolutionary history of the genus. Even though our phylogeny only included four *Batracobdella* species for which genetic data are available. A previous study, which characterises amphibian ectoparasitic leeches from England, reconstructs the evolutionary history of the same four species using fewer number of sequences (Seilern‐Macpherson et al. 2024). Both inferences are overall congruent but show an unsupported discordance in the position of *B. cancricola* (Figure 2A). In our study, *B. cancricola* formed a clade with *B. paludosa*. This clade was the sister group of the clade including *B. reticulata* and *B. algira*. In the previous study, *B. cancricola* is, however, the sister clade of *B. reticulata* and *B. algira*. These two phylogenetic inferences also highlight the high diversity of *B. algira*. The salamander ectoparasitic leeches from the Mediterranean islands of Sardinia and Corsica were the sister group of *B. algira* samples from Tunisia, Algeria, and England (this last one is believed to be introduced) (Seilern‐Macpherson et al. 2024), and together formed the sister clade of the *B. algira* sample from Spain (Trajanovski et al. 2010), accordingly defining at least three divergent *B. algira* lineages. Further studies are needed to better characterise the evolutionary history of both *B. algira* and the *Batracobdella* genus, which is important for understanding not only these parasitic species but also the evolution of their hosts.

The hosts of the ectoparasitic leeches from Sardinia were *Speleomantes* salamanders. In this study, *Batracobdella* parasitism was reported for all five species of the island with abundances that align with the findings of previous studies (i.e., a low prevalence of this host–parasite interaction across Sardinian *Speleomantes*) (Lunghi et al. 2018; Manenti et al. 2016). Hence, we could compare the reconstructed evolutionary relationships of the leeches with those previously inferred for the Sardinian *Speleomantes*. Five studies have inferred the phylogenetic relationships of *Speleomantes* using genetic markers (Carranza et al. 2008; Chiari et al. 2012; Ehl et al. 2019; Stewart and Wiens 2025; van der Meijden et al. 2009). Among the four eastern Sardinian *Speleomantes*, these previous phylogenetic inferences present conflicting relationships and evolutionary discordances (Figure 3). The most recent inference reanalyses all the available genetic data, reconstructing a clade with *S. imperialis* , *S. supramontis* , and *S. flavus* (Stewart and Wiens 2025). This clade was also reconstructed for the ectoparasitic leeches of these closely related host species, potentially unveiling co‐evolutionary processes between hosts and parasites (Figure 2). Other processes that may have contributed to the evolution of the Sardinian ectoparasitic leeches are host‐switching events (Hayward et al. 2021). When only considering the five island species, the previous host phylogenies place *S. genei* as the sister species of the eastern Sardinian *Speleomantes* (Carranza et al. 2008; Chiari et al. 2012; Ehl et al. 2019; Stewart and Wiens 2025; van der Meijden et al. 2009). If hosts and parasites have completely congruent phylogenies due to co‐evolutionary processes in the *Batracobdella* leeches and *Speleomantes* salamanders' system, *S. sarrabusensis* could instead be the sister species of the four other Sardinian species. Nevertheless, given the previous evidence from host phylogenies, the position of *S. genei* ectoparasitic leech could be more plausibly explained by a host‐switching event.

To better understand this co‐phylogeny, the life history of these ectoparasitic leeches should be further characterised. Closer evolutionary ties between hosts and symbionts have been observed in interactions with vertically transmitted mutualistic symbionts (Hayward et al. 2021). For one side, the parasitism by the Sardinian *Batracobdella* leeches does not appear to affect the body condition of their hosts (Lunghi et al. 2018). Several species of leeches are, however, vectors of blood parasites (Jılta et al. 2025). Whether Sardinian salamander ectoparasitic leeches transmit any endoparasites to their hosts remains unexplored, but could have determined the strength of the co‐evolutionary relationships between *Speleomantes* salamanders and *Batracobdella* leeches. On the other side, both the *Speleomantes* hosts and their ectoparasitic leeches exhibit parental care (Kutschera and Wirtz 2001; Oneto et al. 2010; Sawyer 1971; Vági et al. 2022). The transmission of leech offspring probably requires close contact between other host individuals. Leech transmission might occur during the care of the offspring by female salamanders to target more new hosts. This hypothesised phenological synchrony between the host and the parasite, leading to vertical transmission, could partly explain the observed higher probability of parasitised *Speleomantes* females (Lunghi et al. 2018). Nevertheless, this speculation is not supported by previous field observations. Juveniles are rarely parasitised and leeches are more frequently found on large individuals at the cave entrance far from the cave depths sought by females to lay eggs (Lunghi et al. 2018). Additionally, host specificity and parasite dispersal capacity should also be explored, importantly for characterising potential host‐switching events, which might involve more mobile amphibians, such as anurans. Methodological approaches, such as iDNA (invertebrate‐derived DNA extracted from leech gut contents to target vertebrate DNA), could provide valuable information in this regard (Lynggaard et al. 2022), filling this knowledge gap for the Sardinian salamander ectoparasitic leeches.

The evolutionary history and the biogeography of *Speleomantes* salamanders have sparked great research interest. Multiple studies have investigated the different evolutionary processes behind the *Speleomantes* current range, from the disjunct distribution with its American sister group to the colonisation event or events of the Sardinian island (Carranza et al. 2008; Chiari et al. 2012; Ehl et al. 2019; Shen et al. 2016; van der Meijden et al. 2009; Vieites et al. 2007). Despite advances in the understanding of this group's evolution, many questions are still open. The host‐ectoparasite co‐evolutionary hypotheses proposed here supported the previously described clade of *S. imperialis* , *S. supramontis* , and *S. flavus* (Stewart and Wiens 2025). Notwithstanding, the relationships among these three species remain uncertain. Other relationships that remain obscured are the positions of *S. sarrabusensis* , *S. genei* , and the mainland *Speleomantes* species. We speculated about the position of the mainland species given the previous phylogenetic evidence and host‐ectoparasite co‐evolutionary scenarios, which provide complementary evidence regarding *Speleomantes* evolutionary history (Figure 3), accordingly leading to three new evolutionary hypotheses where mainland species could be the sister group of all Sardinian species, or the sister group of the clade of *S. imperialis* , *S. supramontis* , and *S. flavus* , or the sister group of *S. genei* . These hypothesised evolutionary scenarios need to be tested by acquiring and exploring more information about both parasites and hosts.

Future research should be conducted to understand the life history of the Sardinian salamander ectoparasitic leeches and better disentangle the evolutionary processes behind these host–parasite interactions. This could corroborate the host–parasite co‐evolutionary and host‐switching processes proposed here. Sampling efforts should target *S. genei* and *S. supramontis* ectoparasitic leeches, which are underrepresented in sample size in this study, as well as ectoparasitic leeches from other Sardinian amphibians, including the more mobile anurans. These efforts could help identify additional host‐specific clades of *B. algira* from Sardinia. To resolve the intricate evolution of this host–parasite system, genomic data providing faster‐evolving nuclear markers are required from both parasites and parasitised hosts, using for example reduced representation sequencing data, such as transcriptomes (Torres‐Sánchez et al. 2025). Broad evolutionary frameworks should also be considered since changes in other inheritance systems, for example epigenetics, might also contribute to the evolution of the interacting species, as hypothesised for other amphibian‐parasite systems (Torres‐Sánchez 2025).

## Author Contributions

**María Torres‐Sánchez:** conceptualization (lead), data curation (lead), formal analysis (lead), investigation (lead), methodology (lead), visualization (lead), writing – original draft (lead), writing – review and editing (lead). **Michael Veith:** investigation (supporting), project administration (supporting), supervision (supporting), writing – review and editing (supporting). **Enrico Lunghi:** conceptualization (supporting), investigation (supporting), project administration (lead), supervision (lead), writing – review and editing (supporting).

## Conflicts of Interest

The authors declare no conflicts of interest.

## Supporting information


**Data S1:** ece373019‐sup‐0001‐Supinfo.pdf.

## Data Availability

The newly generated COI and 16S sequences are available in the NCBI GenBank database (see Table S1 for GenBank IDs).
