# Supplementary material for: Can Ectoparasite Phylogenetics Shed Light on Host Evolution? The Batracobdella Leeches and Speleomantes Salamanders' System
Source: Ecol Evol. 2026 Feb 15;16(2):e73019. doi: 10.1002/ece3.73019 (PMC12906977; doi:10.1002/ece3.73019)
Supplement: Supplementary file 1 — Data S1: ece373019‐sup‐0001‐Supinfo.pdf. [file ECE3-16-e73019-s001.pdf]

SUPPLEMENTARY INFORMATION

Can ectoparasite phylogenetics shed light on host evolution? The *Batracobdella* leeches and *Speleomantes* salamanders' system

María Torres-Sánchez[1*], Michael Veith[2], Enrico Lunghi[1]

[1]Department of Life, Health, and Environmental Sciences, University of L'Aquila, L'Aquila, Italy
[2]Biogeography Department, Trier University, Trier, Germany

[*]Corresponding author: María Torres-Sánchez (torressanchez.maria@gmail.com)

This file includes:

Tables S1 and S2

Figure S1

Table S1. Information regarding the leech sequences generated in this study. Table shows the barcode region: the cytochrome C oxidase subunit I (COI) or the small subunit ribosomal RNA gene (16S), the host for the ectoparasitic leeches, the sampling region, and its GenBank ID. The table also indicates the sequences used (Y) in the Shimodaira-Hasegawa test (SH test).

Sequence name	Gene	Host	Location	Sequences for SH test	GenBank ID
Sflavus_leech_1	COI	*Speleomantes flavus*	Sardinia		PX860335
Sflavus_leech_3	COI	*Speleomantes flavus*	Sardinia		PX860336
Sflavus_leech_5	COI	*Speleomantes flavus*	Sardinia		PX860337
Sflavus_leech_9	COI	*Speleomantes flavus*	Sardinia		PX860341
Sflavus_leech_10	COI	*Speleomantes flavus*	Sardinia		PX860342
Sflavus_leech_11	COI	*Speleomantes flavus*	Sardinia	Y	PX860343
Sflavus_leech_12	COI	*Speleomantes flavus*	Sardinia		PX860344
Simperialis_leech_6	COI	*Speleomantes imperialis*	Sardinia		PX860338
Simperialis_leech_7	COI	*Speleomantes imperialis*	Sardinia		PX860339
Simperialis_leech_8	COI	*Speleomantes imperialis*	Sardinia		PX860340
Simperialis_leech_19	COI	*Speleomantes imperialis*	Sardinia		PX860351
Ssupramontis_leech_15	COI	*Speleomantes supramontis*	Sardinia	Y	PX860349
Ssarrabusensis_leech_13	COI	*Speleomantes sarrabusensis*	Sardinia		PX860345
Ssarrabusensis_leech_14	COI	*Speleomantes sarrabusensis*	Sardinia		PX860346
Ssarrabusensis_leech_17	COI	*Speleomantes sarrabusensis*	Sardinia	Y	PX860350
Sgenei_leech_20	COI	*Speleomantes genei*	Sardinia	Y	PX860348
Emontanus_leech_1_a	COI	*Euproctus montanus*	Corsica	Y	PX860347
Free-living_leech_1	COI	-	Sardinia		PX860352
Free-living_leech_2	COI	-	Abruzzo		PX860353
Sflavus_leech_1	16S	*Speleomantes flavus*	Sardinia		PX695470
Sflavus_leech_2	16S	*Speleomantes flavus*	Sardinia		PX695471
Sflavus_leech_4	16S	*Speleomantes flavus*	Sardinia		PX695472
Sflavus_leech_9	16S	*Speleomantes flavus*	Sardinia		PX695475
Sflavus_leech_10	16S	*Speleomantes flavus*	Sardinia		PX695476
Sflavus_leech_11	16S	*Speleomantes flavus*	Sardinia		PX695477
Sflavus_leech_12	16S	*Speleomantes flavus*	Sardinia		PX695478
Simperialis_leech_7	16S	*Speleomantes imperialis*	Sardinia		PX695473
Simperialis_leech_8	16S	*Speleomantes imperialis*	Sardinia	Y	PX695474
Simperialis_leech_19	16S	*Speleomantes imperialis*	Sardinia		PX695479
Free-living_leech_1	16S	-	Sardinia		PX695480
Free-living_leech_2	16S	-	Abruzzo		PX695481

Table S2. Results of the Shimodaira-Hasegawa test. Likelihood (L) of the two tree topologies (parasites and hosts) fitted to the alignment of parasite sequences.

Tree	lnL	Diff lnL	p-value
Parasite topology	-1314.329	0	0.491
Host topology	-1319.281	4.952	0.268

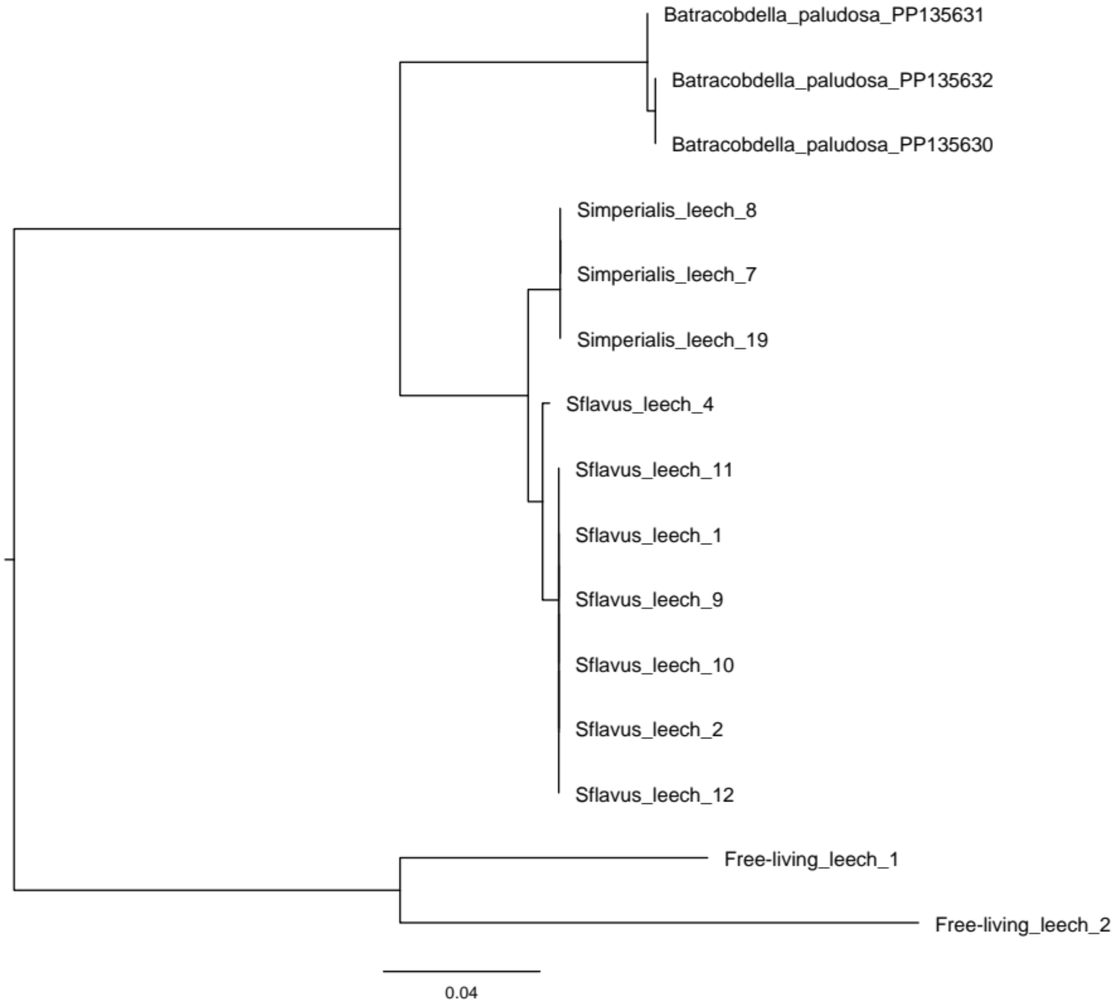

Figure S1. 16S phylogeny of *Batracobdella* leches. Inferred tree using publicly available 16S sequences for the genus and samples from two *Speleomantes* hosts (*S. flavus* and *S. imperialis*).
